# Reconstruction and Visualization of Human Gastrointestinal Tract

**Published:** 2012-03

**Authors:** Rong-guo Yan, Xu-dong Guo, Changqing Xu

**Affiliations:** 1 *School of medical instrument and food engineering, University of Shanghai for Science and Technology, Shanghai, China;*; 2 *Shidong hospital of Shanghai, Shanghai, China*

**Keywords:** gastrointestinal tract, marching cubes algorithm, surface reconstruction, three-dimensional visualization

## Abstract

**Background:**

Converting the two-dimensional (2D) cross-sectional photographs into an intuitive three-dimensional (3D) model is a basic task for medical imaging data for auxiliary disease-linked diagnosis purpose.

**Methods:**

Reconstruction and visualization process of gastrointestinal cross-sectional photographs includes image preparation, image registration, image segmentation, 3D surface-rendering reconstruction, and implementation of 3D digital visualization.

**Results:**

Using the visualization toolkit (VTK), we implemented 3D digital reconstruction and visualization of gastrointestinal tract, whose visualized model can be zoomed, paned, and rotated, including the stomach, the small intestine, and the large intestine.

## INTRODUCTION

As we know, human gastrointestinal tract, another broader term of which is the digestive system, refers to all the structures from the mouth to the anus. It consists of the upper parts including oesophagus, stomach and duodenum, and the lower parts including most of the small intestine and all of the large intestine, sometimes the anus is also included.

On the other hand, thanks to the development of modern medical imaging technology like X-ray computed tomography (CT) (1), magnetic resonance imaging (MRI) (2) etc., clinical examination and diagnosis of human diseases have been brought an evolutionary innovation. These imaging systems can really create rich and colourful cross-sectional photographs of tissues and organs. The doctors usually estimate the size and shape of the pathological area of interest (AOI) of the disease by their experiences through series of two-dimensional (2D) consecutive cross-sectional photographs, and imagine their three-dimensional (3D) geometrical relationship of the pathological change spot with the surrounded tissues in their minds, which is sometimes difficult to be imagined since this process is too abstract.

In order to improve accuracy of medical diagnosis and treatment, transforming 2D cross-sectional photographs to an intuitive stereoscopic image, exhibiting human organs’ 3D structure and shape, and consequently providing certain anatomical structure information, which is unable to be obtained by the traditional method, become the present multi-disciplinary overlapping hot spot. Under this background, many researchers and doctors have tried many methods to realize 3D reconstruction and visualization of such medical imaging photographs, and have achieved many good results.

In General, it is easy to obtain 2D cross-sectional colourful photographs for human surface organs. However, it is rather difficult to obtain such 2D cross-sectional photographs for *in vivo* human organs like gastrointestinal tract as an example for its unreachable, hollow tube-like and complex structures. In the paper, we used the gastrointestinal photographs from the U.S. Visible Human Project (VHP) to perform 3D reconstruction and visualization (3).

## METHODS

### Three-dimensional Reconstruction and visualization

**Figure 1:**
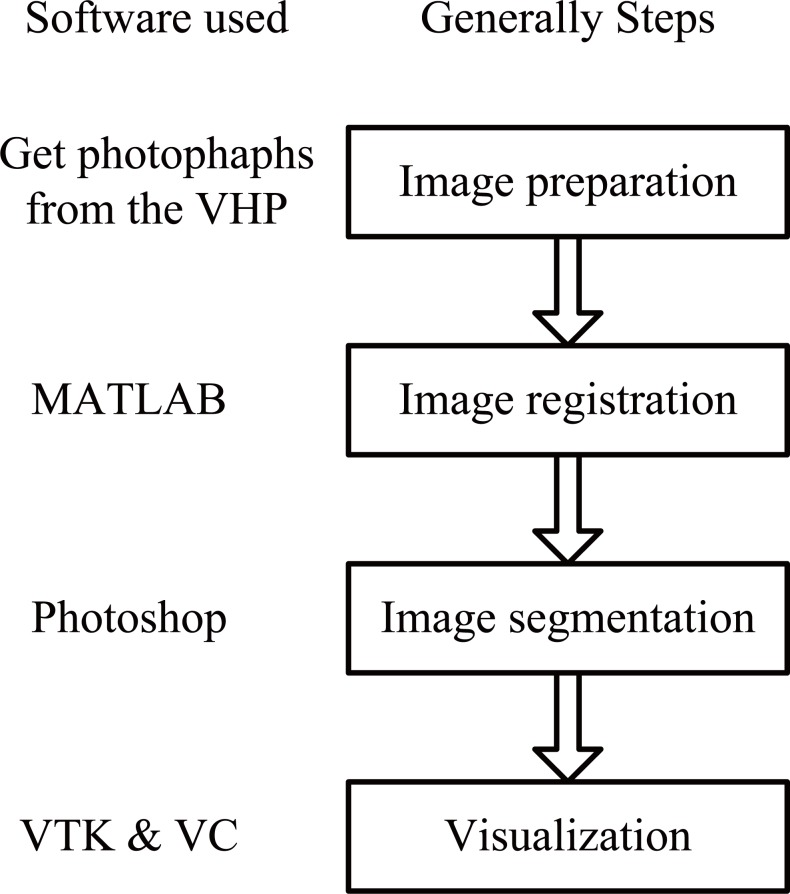
Data processing flow chart. Figure 1 shows general processing flow chart of 3D reconstruction and visualization, which includes image preparation, image registration, image segmentation and visualization. The software that we used is listed in the left of the flow chart.

Three dimensional reconstruction and visualization of medical photographs consisted of the following four steps as shown in the Figure 1. 1) First step was to get the cross-sectional photographs (i.e., image sequence or image slices) and made some preparation work to the cross-sectional photographs, like filtering and enhancement, etc. Usually the photographs obtained were 2D cross-sectional ones with a certain storage format, portable network graphic (PNG) format for example, which needed to make certain format conversion, so as to facilitate data reading, reconstruction and visualization programming by the software; 2) Next step was image registration. When performing taking cross-sectional slice photographs, the position, angle and other parameters of the camera might be altered due to some human factors, the photographs obtained needed to carry on certain image registration processing. In the paper, we used MATLAB to accomplish such image registration based upon the feature point detection method; 3) The image segmentation was to get those medical parts of interest. In the paper, we segmented gastrointestinal segment from 2D cross-sectional scanning slices by hand using the Photoshop software because that the gastrointestinal tissue boundaries are rather obscure and sometimes geometrical adhesive making it difficult for automatic segmentation; 4) Finally, we adopted certain software to implement graphical model reconstruction, visualization and interaction operations like dynamic zoom, pan, rotation and other interactive operations. In the paper, we used the visualization toolkit (VTK) embedded in Microsoft Visual Studio C++ (VC6) to accomplish three-dimensional reconstruction and visualization.

**Preparation of the image photographs.** The cross-sectional photographs used in the paper for three-dimensional reconstruction and visualization are from the visible human male data set, which is run by the University of Michigan visible human project (VHP) (3). The VHP is an effort to create a detailed data set of cross-sectional photographs of the human body, in order to facilitate anatomy visualization applications.

In the paper, the cross-sectional photographs were from the male 38-year-old cadaver, who was encased and frozen below zero 80 degrees Celsius in a gelatin and water mixture in order to stabilize the specimen for cutting. The specimen was then ‘cut’ (grinding away the top surface of a specimen at regular intervals) in the axial plane at 1 mm intervals. Each of the resulting 1,871 ‘slices’ (the revealed surface of the specimen to be photographed) were photographed in digital. The approximately 7.5 megabyte axial anatomical images were 2048 pixels by 1216 pixels, with each pixel being 0.33mm in size, and defined by 24 bits of colour.

**Image registration.** Due to different imaging conditions when data acquisition was performed, there were differences between the sensed images and the reference image. And, image registration, is just the process of overlaying two or more images of the same scene taken at different times, from different viewpoints. The aim of image registration was to geometrically align the reference and sensed images.

**Figure 2:**
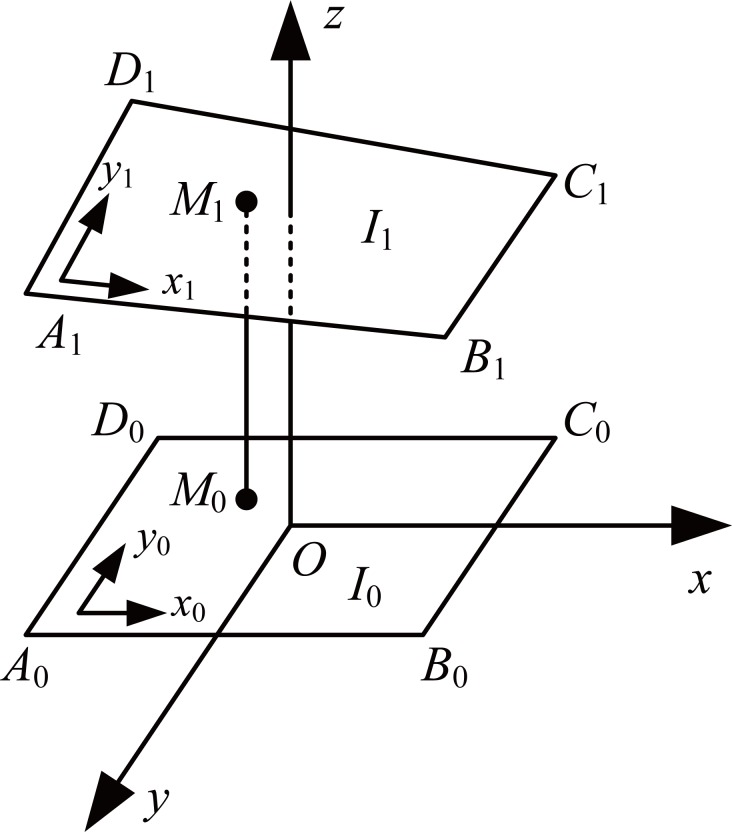
Principle of image registration. Figure 2 illustrates the principle of image registration. In the figure, *I*_1_ is the image to be registered, and *I*_0_ is the reference image.

Figure 2 illustrated the principle of image registration. In the figure, *I*_1_ was the image to be registered, and *I*_0_ was the reference image. Then, the relationship between any point *M*_1_(*x*_1_,*y*_1_,*z*_1_) in the *I*_1_ and the point *M*_0_(*x*_0_,*y*_0_,*z*_0_) at the identical position in the *I*_0_ could be expressed as,

(a) x1y1z1=T x0y0z0,T=t11t12t13t21t22t23t31t32t33

Three dimensional transformation matrix *T* could be calculated by multiple marked feature points (the VHP used three feature points, i.e., three separate steel alignment rods) in the sliced images. In the paper, we used image registration tool in the image processing toolbox (IPT) in MATLAB to accomplish the process of image registration of the sliced images using the feature point detection method.

**Image segmentation.** Image segmentation referred to the process of partitioning a digital image into multiple segments to locate objects and boundaries (lines, curves, etc.) that we were interested in. The result of image segmentation was a set of segments that collectively cover the entire image, or a set of contours extracted from the image.

Due to geometrical adhesion, structural complexity, as well as similar colour between the peripheral tissue, especially between the mesentery, the fat and etc. of the gastrointestinal tract in the slices, it was hard to find a rule to do automatic segmentation of such images using a certain algorithm. Therefore, in the paper, we adopted a magnetic lasso tool in the Photoshop software to accomplish image segmentation and saved them as the *.jpg format, so that the VTK software could carry on image reading operation, reconstruction and corresponding 3D visualization with interactive operations.

**Basic theory of marching cubes.** The method used for three-dimensional reconstruction in the paper was the marching cubes (MC) algorithm (4).

Marching cubes, which was published in the 1987, is a computer graphics algorithm for extracting a polygonal mesh of an isosurface from a three-dimensional volume. The isosurface here represents points of a constant value within a volume of space.

As the name suggests, marching cubes only works with voxels (i.e., cubic-cells). By analyzing voxel individually, you can quickly and easily determine if the eight corner points are “above” or “below” the desired isovalue (the isovalue is the contour value for the isosurface). Each of these 8 values can easily be mapped to a single bit of an 8-bit number. Let the *i*-th vertex of a voxel is available as *P*(*x_i_,y_i_,z_i_,q_i_*), *x_i_, y_i_, z_i_* are the coordinates of point *P* in the Cartesian coordinate system, *q_i_* is a physical property of the value of point *P* (in fact, the iso-points of the isosurface are those points with the same attribute value.), and the state value *S* of the *i*-th vertex to a given attribute value *C*_0_ extracted from the isosurface can be expressed as,

(b) $$S=\left\{\begin{array}{c} 1\\ 0 \end{array}\begin{array}{ccc} q_{i} & \geq & C_{0}\\ q_{i} & < & C_{0} \end{array}\right.$$

**Figure 3:**
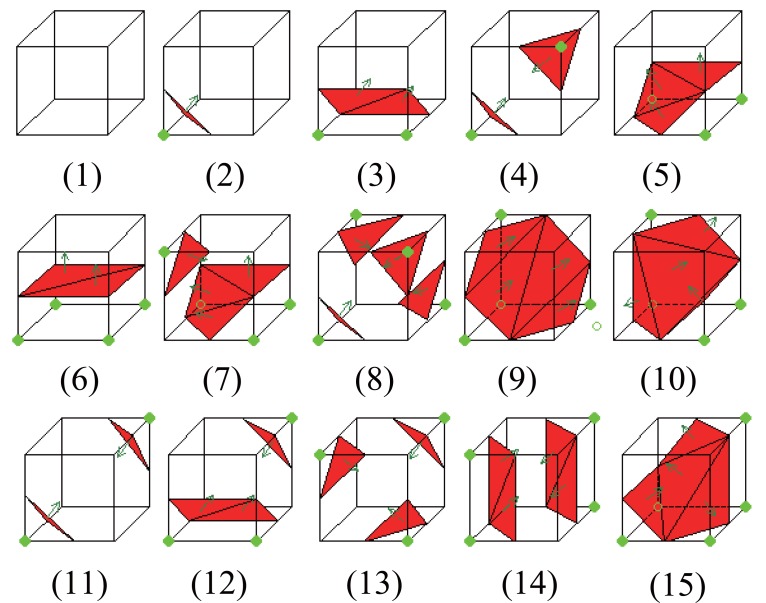
Example of the 15 unique MC Solutions. Figure 3 shows 15 unique solutions to the marching cubes, reduced from all 255 combinations using geometric symmetric.

There are 2^8^-1=255 possible combinations to the problem as showed in the Figure 3. These all 255 combinations can be further reduced, using geometric symmetric, down to 15 unique solutions as the Figure 3 shows.

Most implementations to create points along the edges at this point use a simple lookup table and linear interpolation. Let two points are *P_i_*(*x_i_,y_i_,z_i_,q_i_*) and *P_j_*(*x_j_,y_j_,z_j_,q_j_*), according to the theory of linear interpolation, the iso-point P(*x,y,z*), can be obtained,

(c) $$\left\{\begin{array}{c} x=x_{i}+Q\left(x_{j}-x_{i}\right)\\ y=y_{i}+Q\left(y_{j}-y_{i}\right)\\ z=z_{i}+Q\left(z_{j}-z_{i}\right) \end{array}\right.$$

(d) $$Q=\left(q_{i}-C_{0}\right)\left(q_{j}-C_{0}\right)$$

After those iso-points are computed on the voxel edges, we can connect these iso-points into triangles or polygons to form parts of the isosurface.

In order to show the image of such isosurface, vertex normals must be given, and can be computed using the gradient (derivative of the density function) between the vertices, interpolated along the voxel edges using central differences along the three coordinate axes by,

(e) $$\left\{\begin{array}{c} g_{x}\left(i,j,k\right)=\frac{D\left(x_{i+1},y_{j},z_{k}\right)-D\left(x_{i-1},y_{j},z_{k}\right)}{\Delta x}\\ g_{y}\left(i,j,k\right)=\frac{D\left(x_{i},y_{j+1},z_{k}\right)-D\left(x_{i},y_{j-1},z_{k}\right)}{\Delta y}\\ g_{z}\left(i,j,k\right)=\frac{D\left(x_{i},y_{j},z_{k+1}\right)-D\left(x_{i},y_{j},z_{k-1}\right)}{\Delta z} \end{array}\right.$$

where *D*(*x_i_, y_j_, z_k_*) is the density at pixel (*i, j*) in slice *k.* Δ*x*, Δ*y*, Δ*z* are lengths of the cube edges.

**3D reconstruction and visualization.** From the set of 2D segmented slices produced by the former step, we, then, computed a 3D model of the GI parts of interest for visualization purposes. Thanks to a discrete 3D smoothing algorithm, we computed interpolated slices between the original ones. This method allowed computing a geometrical model which was homogeneous in each dimension, despite the difference between the pixel resolution in each slice and the interslice distance. We computed a 3D model of each part using a topologically sound variant of the marching cubes method (5, 6). These models were actually represented by closed surfaces composed of triangles (mesh representation). They could thus be visualized through standard rendering tools to make a mesh representation of 3D objects. From these models, many post-processing analysis, computing the volumes of different tissues, analysis on stress and strain based upon the finite element, and etc., might be implemented.

### Introduction to the VTK

The visualization toolkit (VTK) was an open-source, freely available software system for 3D computer graphics, image processing and visualization (7-10). The VTK, consisted of a C++ class library, integrated a wide variety of visualization algorithms and advanced modelling techniques, which made it easy and possible for our medical visualization.

**Figure 4:**
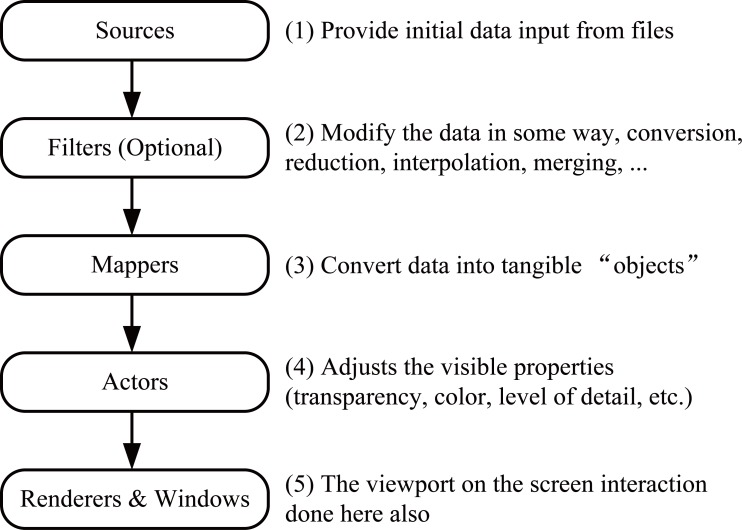
VTK visualization pipeline. Figure 4 shows the VTK visualization pipeline, whose main components are sources, filters, mappers, actors, and renderers & windows.

The central structure of the VTK was a pipeline of data, from a source of information to an image rendered on the screen as seen in the Figure 4. The main components were *sources, filters, mappers, actors*, and *renderers & windows*. 1) Sources were quite simply the source of data flowing through the visualization pipeline; 2) *Filters* were VTK components that received data from other components, modified the data in some way, and then delivered the modified data as output to be used by other components. Filters might extract some portion of a large data set, sub-sample data sets to coarser resolution, interpolate data sets to a finer resolution, merged multiple inputs into a combined output, split compound inputs into component parts, or a wide variety of other transformations; 3) *Mappers* were the VTK components that received data from other components, (usually filters, but sometimes directly from sources), and “mapped” the data to some sort of a physical manifestation that could be rendered by the rendering engine; 4) *Actors* were the VTK components that allowed for the adjustment and control of the appearance properties of the physical manifestations of the data as rendered onto the screen. Some of the properties typically controlled via actors were transparency and color mapping; 5) *Renderers & Windows* represented the end of the VTK pipeline, which users actually saw on the screen.

## RESULTS

**Figure 5:**
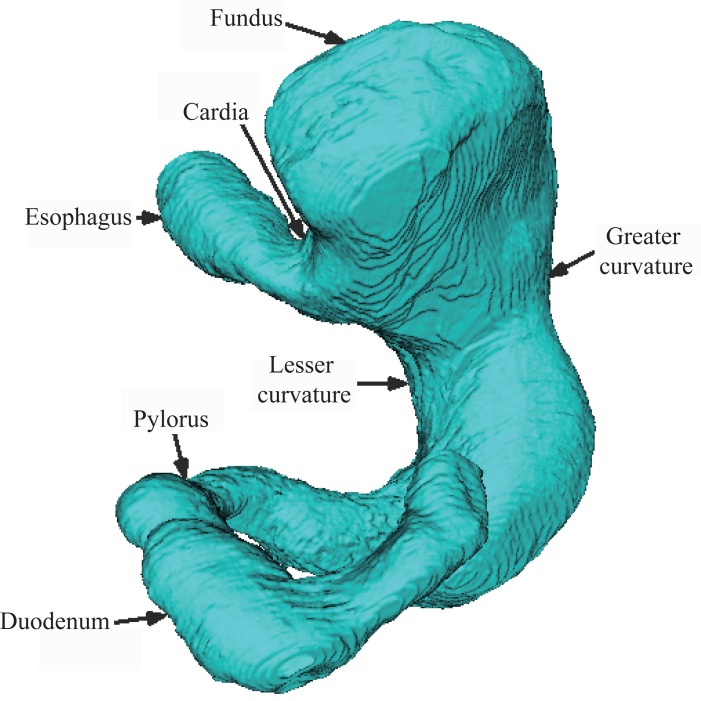
Reconstruction of the stomach. Figure 5 shows reconstructed model of the stomach. From the figure, the fundus, the cardia, the greater curvature, the lesser curvature, the pylorus of the stomach, as well as the esophagus and the duodenum are clearly seen.

**Figure 6:**
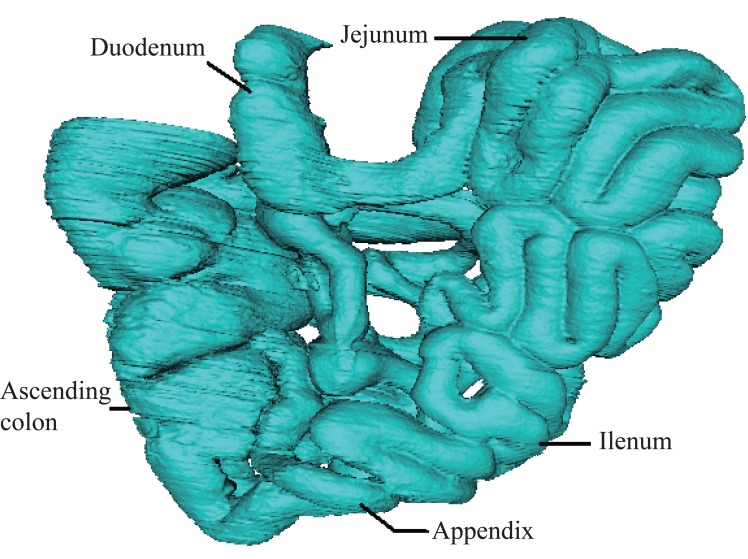
Reconstruction of the small intestine. Figure 6 shows the reconstructed small intestinal tract. The small intestine with an irregular shape, twisting and turning around between the stomach and the large intestine, is divided into three structural parts including duodenum, jejunum and ileum.

**Figure 7:**
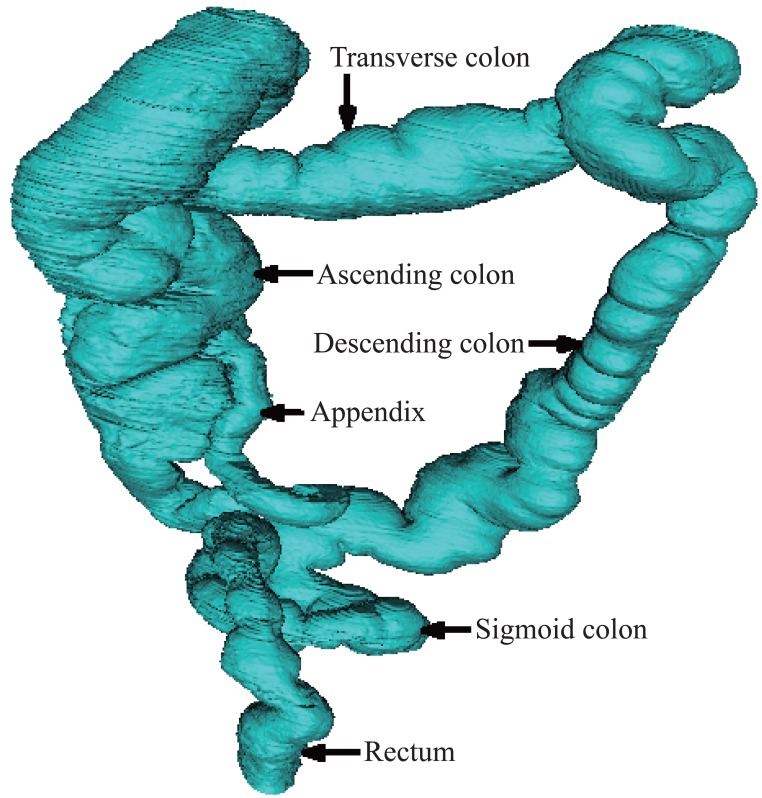
Reconstruction of the large intestine. Figure 7 shows the reconstructed large intestine. The locations along the colon are the ascending colon, the transverse colon, the descending colon and the sigmoid colon.

Using the visualization steps introduced above and the VTK software, we have carried on three dimensional reconstructions to GI tract including the stomach, the small intestine, and the large intestine as shown in the Figure 5, in the Figure 6 and in the Figure 7.

## DISCUSSION AND CONCLUSIONS

Reconstructed model of the stomach was showed in the Figure 5. From the figure, we can clearly see the fundus, the cardia, the greater curvature, the lesser curvature, the pylorus of the stomach, as well as the esophagus and the duodenum. As we know, the stomach is an organ of digestion. It has a sac-like shape and is located between the esophagus and the small intestine. Due to eating no food, the stomach of this male is empty, and its volume is rather small, which can also be seen on the consecutive photographs.

Reconstructed small intestinal tract is shown in the Figure 6. The small intestine is the part of the gastrointestinal tract following the stomach and followed by the large intestine, and is where much of the digestion and absorption of food takes place. As seen in the figure, the small intestine with an irregular shape, twisting and turning around between the stomach and the large intestine, is divided into three structural parts including duodenum, jejunum and ileum.

The large intestine is the second-to-last part of the digestive system with function to absorb water from the remaining indigestible food matter, and then to pass useless waste material outside the body. As shown in the Figure 7, the locations along the colon are the ascending colon, the transverse colon, the descending colon and the sigmoid colon.

## FUTURE WORKS

Due to work pressure and fast pace of modern life, people’s present diet structure and life style have had big changes. For some known or unknown factors, people who suffered from chronic constipation, fecal impaction or lower intestinal obstruction became more and more, especially in middle-aged and elderly persons. Such diseases gave much pain to the patients, and might cause colorectal cancer when the chronic constipation became worse. Colorectal hydrotherapy (i.e., water cure or therapy) was a good method by using water to relief pain and to treat such illness. Magnetized warm water was repeatedly slowly infused into colorectal section via the rectum to dilute, soften and remove stool feces, non-specific toxins from the colon and intestinal tract, so that they could be discharged timely out of the body. However, how to control the temperature, pressure and velocity of the colorectal hydrotherapy equipment was rather difficult. If the water pressure was higher than 100mbar, it might cause intestinal perforation. During the process of treatment, another question was the intestine might involuntarily contract due to emotional tension and made the treatment ineffective.

Based on the existing VTK gastrointestinal digital 3D visualization model, finite element post-processing system based on the VTK software could be developed. This system could give numerical simulation of water pressure, water temperature, water velocity and colorectal deformation, and give feasible proposals for such disease treatment and health care.

Moreover, such 3D visualization model could be used for researches on 3D geometric structural distribution and motion dynamics of the gastrointestinal tract, and etc.
